# Management of Clamshell Fractures in Total Hip Arthroplasty: A Rarely Recognized Periprosthetic Injury Pattern

**DOI:** 10.3390/jcm14144896

**Published:** 2025-07-10

**Authors:** Felix Haussner, Michael Fuchs, Moritz Oltmanns, Heiko Reichel, Tobias Freitag

**Affiliations:** Department of Orthopaedic Surgery, University of Ulm, Oberer Eselsberg 45, 89081 Ulm, Germany; michael.fuchs@rku.de (M.F.); moritz.oltmanns@rku.de (M.O.); heiko.reichel@rku.de (H.R.); tobias.freitag@rku.de (T.F.)

**Keywords:** periprosthetic fracture, clamshell fracture, total hip arthroplasty, Unified Classification System, Vancouver classification, open reduction internal fixation, femoral fracture, hip replacement

## Abstract

Periprosthetic femoral fractures (PPFFs) represent the third most frequent indication for revision total hip arthroplasty (THA). Given the steadily increasing number of primary hip replacements, this complication is gaining growing attention among orthopedic surgeons. Clamshell fracture (CF) constitutes a particularly controversial and underrecognized fracture pattern that, for a long time, was not accounted for in the commonly used classification systems for PPFFs. Recent studies suggest that the incidence and clinical relevance of these injury patterns have been underestimated. Therapeutic options are manifold and depend on various patient-specific factors as well as stem stability. Despite this, the current literature remains limited, and standardized therapeutic approaches are still poorly defined. This review aims to provide a comprehensive overview of clamshell fractures as a distinct pattern of periprosthetic injuries. Furthermore, relevant treatment options dependent on biomechanical considerations will be outlined and discussed.

## 1. Introduction

Periprosthetic femoral fractures (PPFFs) represent a serious complication associated with considerable morbidity and significant socioeconomic impact [1]. In Germany, the number of statistically recorded primary THA procedures increased from 152,584 in 2007 to 187,640 in 2023 [2]. With the growing number of primary arthroplasties, consecutive complications following THA will also rise in number. With regard to the technical aspects of primary THA, there is a notable trend toward an increasing use of uncemented femoral stems over the years [3,4]. However, numerous studies suggest that cementless stems are associated with a higher risk of fracture [5,6,7,8,9,10]. Hence, the growing clinical relevance of PPFFs, particularly in an aging population, necessitates a deeper understanding of these conditions and their respective management strategies. As such, anatomically shaped and metaphyseal-anchoring femoral stems are suspected to increase the risk of so-called clamshell fractures, a distinct fracture pattern involving the lesser trochanter and the proximal segment of the medial femoral wall with preservation of the lateral cortex [9,11,12]. This characteristic morphology involves unique biomechanical and therapeutic considerations. Of note, the biomechanical fracture mechanism is aligned to medial cortical overload, primarily associated with metaphyseal-anchored stem designs. The clamshell fracture pattern was first described by Mallory et al. in 1989 as an ostensibly intraoperative complication [13]. However, Capello et al. later defined these lesions as postoperative complications [12]. Since the CF was not included as a distinct entity in the widely used Vancouver classification system (VCS), Capello et al. suggested a modified version by recognizing CF as a separate entity [12]. In 2018, Huang et al. further modified the Unified Classification System (UCS) published by Duncan and Haddad in 2014 [14] as a continuation of the VCS, by defining CF as a separate subtype of periprosthetic femoral fractures [15]. Recent studies suggest that these injury patterns may be more common and clinically significant than previously assumed [11,12,16,17].

Despite increasing recognition, the literature on clamshell fractures remains limited, and treatment strategies are not yet well established. The objective of this narrative review is to provide a structured overview of the epidemiology and risk factors, classification, biomechanical considerations, and current therapeutic options related to this fracture type.

## 2. Epidemiology and Risk Factors

PPFFs represent the third most common indication for revision surgery following total hip arthroplasty [1]. However, the reported incidence of intraoperative PPFFs varies widely in the literature, and data on clamshell fractures remain limited and inconsistent. Capello et al. reported a rate of 0.9% in their series of 1039 cementless hip prostheses, with an overall incidence of PPFFs of 5.6% [12]. All CFs occurred postoperatively, with 67% presenting within the first 2 months after surgery [12]. Egrise et al. analyzed 445 cases of PPFFs and identified clamshell fractures in 33 patients (7.5%). Notably, these fractures occurred in both the early and late postoperative courses and were specifically associated with cementless femoral stems [16]. Karam et al. found a clamshell pattern in 30 of 172 PPFFs (17%), with a significant correlation to cementless wedged and straight-stem designs; only 1 case was observed in a cemented prosthesis [17]. In a registry-based study by Cottino et al., involving 3593 implantations, the reported PPFF incidence was 1.25% at a mean follow-up of 6 years, with 44% of these fractures classified as CFs [11]. In this study, CFs were significantly increased in cementless anatomical stems compared to straight stems [11]. In a consecutive series that included only cementless short-stem THAs, Staunton et al. reported a PPFF incidence of 0.6% in 3192 implantations, with 26% of fracture types classified as CFs [6]. All cases in this study occurred postoperatively within the first 6 weeks [6].

Primarily, it is essential to highlight the general risk factors associated with PPFFs. Female sex and age over 65 years have been identified as independent risk factors for the occurrence of PPFFs, presumably confounded by osteoporosis [5,18]. Additional patient-related risk factors include rheumatoid arthritis, Paget disease, obesity, and other comorbidities, such as a history of malignancy [11,18,19]. Reduced bone mineral density has been shown to increase the risk of intraoperative femoral fractures by a factor of five [18]. Nevertheless, reduced bone mineral density increases the risk of hip fractures. The latter are often treated with cemented or cementless THA and pose a certain threat for consecutive PPFFs [20]. Notably, uncemented femoral components are associated with a 14-fold increased risk of PPFF compared with cemented stems [10]. The risk of PPFF does not plateau after the early postoperative period but continues to increase over time [10]. Particularly in cases of poor bone quality, a risk reduction to prevent PPFF can be achieved by using a cemented implant. In addition to the general risk factors, intraoperative surgical technique—particularly during stem implantation—plays a crucial role in the development of clamshell fractures. Notably, shortened conventional stem designs may contribute to medial femoral cortex overload due to their design and frequent varus positioning [6]. Furthermore, several authors reported that a relevant proportion of clamshell fractures occur intraoperatively during broaching and stem insertion [1,6,11]. Additionally, CFs can happen in the early postoperative period as a result of caudal stem migration by using a primary metaphyseal anchored stem [6,7]. To date, the literature is inconsistent regarding whether femoral cementless short-stem or straight-stem designs are associated with a lower overall risk of PPFFs [21]. Targeted investigations addressing the impact of stem design on the occurrence of clamshell fractures are limited and will be discussed in a subsequent chapter.

## 3. Towards Improved Fracture Classification—The Role of Clamshell Fractures in VCS and UCS

**Figure 1:**
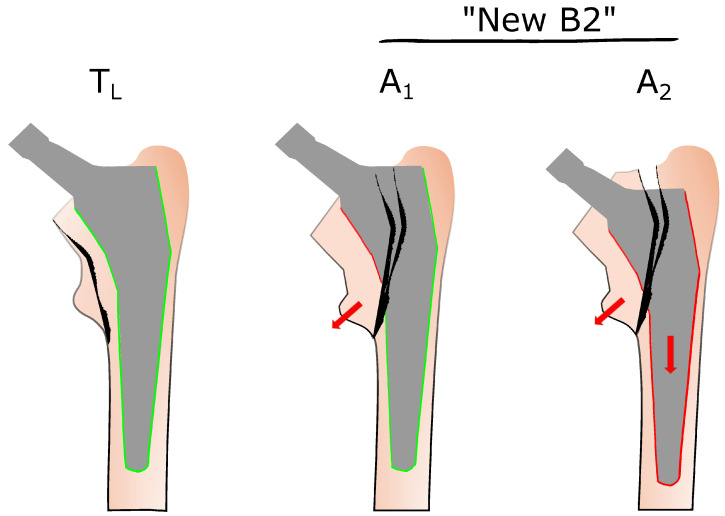
Modified Vancouver classification system for clamshell fractures (A_1_ and A_2_), as described by Capello et al. [12]. Fractures involving the lesser trochanter are designated as T_L_, indicating no compromise of stem stability. The classical “new B2 fracture” is further subdivided into A_1_ (with a stable stem) and A_2_ (with a loose stem). Typically, the A_2_ fracture pattern is associated with stem subsidence. Red arrow: direction of minor trochanteric dislocation. Color lines around the stem illustrate stem fixation: green line: fixed stem, red line: loose stem.

To differentiate between commonly encountered PPFFs, the VCS was initially developed by Duncan and Masri in 1995 [8]. It remains the most widely adopted system for the treatment of femoral fractures after total hip arthroplasty. The VCS is considered straightforward, reliable, and clinically valuable, as it provides guidance for therapeutic decision making based on specific fracture characteristics. It is based on three key criteria, namely the location of the fracture, implant stability, and the quality of the surrounding bone stock [22,23]. In general, treatment algorithms for PPFFs are closely aligned with the Vancouver classification types. Historically, the VCS did not differentiate between a simple avulsion of the lesser trochanter (type A_LT_) and a more serious fracture involving the medial femoral cortex, which can compromise stem stability [11,12]. Vancouver type A fractures are subcategorized into A_GT_ (greater trochanter) and A_LT_ (lesser trochanter) types, but both are defined by a well-fixed stem. Type A_LT_ fractures may result from traction forces of the iliopsoas muscle, with the lesser trochanter serving as the site of avulsion. Generally, isolated type A_LT_ fractures are rare and typically associated with osteoporotic bone or osteolysis [24]. These fractures do not compromise the stability of the femoral stem, and the therapeutic approach is mainly conservative [24]. The anatomical localization near the lesser trochanter may initially suggest that clamshell fractures should be classified as Vancouver type A lesions. However, their fracture pattern necessitates classification as a type B fracture because the bone defect extends to involve the proximal medial femoral wall, and stem stability may be at risk [9]. Therefore, it is crucial to distinguish between isolated avulsion fractures of the lesser trochanter and more extensive fracture patterns that may compromise stem stability because subsequent therapeutic approaches differ substantially. The avulsion of the lesser trochanter along with involvement of the proximal medial cortex and subsequent destabilization of the stem was first described as a clamshell fracture by Van Houwelingen and Duncan in 2011 [9]. To integrate CFs within the VCS, the fracture type was defined as “new B2,” which implies that all CFs are inherently unstable. In contrast, Capello et al. conducted a prospective analysis of PPFFs and found that CFs were associated with both stable (A_1_) and loose (A_2_) femoral stems, as illustrated in Figure 1 [12]. Therefore, they investigated 1032 hips and identified 58 PPFFs, corresponding to an incidence of 5.6%. Notably, 9 of 20 postoperative PPFFs were not classifiable using the conventional VCS [12]. Consequently, a modified VCS was proposed, reclassifying A_LT_ and A_TG_ fractures as T_L_ and T_G_, respectively, and CFs as A_1_ and A_2_ (Figure 1) [9]. It should be emphasized at this point that the declaration of CFs with the labels A_1_ and A_2_ is potentially misleading, as it may imply that CFs are type A fractures. To incorporate the injury pattern of clamshell fractures in a commonly used classification system, Huang et al. modified the UCS in 2018 [15]. Clamshell fractures are recognized as a distinct entity under the label “B2PALT”, which includes the destabilization of the stem.

## 4. Biomechanical Considerations

As mentioned above, CFs may occur both intraoperatively and during the postoperative period [6,9,12,16,25]. The appropriate selection of stem design and implant size, adapted to an individual’s anatomy, significantly impacts the occurrence of PPFFs, including CFs [17]. Many modern cementless femoral stem designs aim to achieve metaphyseal fixation with proximal load transfer to approximate physiological load distribution [6,25]. Clamshell fractures involve the medial femoral cortex, including the residual neck, calcar, and lesser trochanter. These anatomical areas are critical for achieving metaphyseal stability. Consequently, the required structural integrity for stable metaphyseal stem fixation is frequently compromised in the presence of these fractures [26]. In femoral stems with both metaphyseal and diaphyseal fixation or exclusively diaphyseal anchorage, stem stability depends on the extent of compromised bone in the proximal medial cortex. In contrast, implants with mainly metaphyseal anchorage rarely maintain their stability when a CF is present. In a biomechanical experiment using diaphyseal anchoring press-fit stems in human cadaveric femora, the effect of distal cerclage banding relative to a CF on stem stability was investigated. The study demonstrated that intraoperative CFs involving up to 40% of the diaphyseal anchoring zone could be successfully stabilized under dynamic loading conditions using distal cerclage banding [7].

Clamshell fractures are more commonly observed in anatomically shaped and wedge-designed uncemented implants [9,11,12,17,22]. A recent study demonstrated that CFs also occur during the early postoperative period following cementless short-stem THA [6]. Unrecognized intraoperative fractures have also been discussed as a cause of early postoperative CFs. Notably, the abovementioned study involved shortened conventional stem designs rather than curved short stems, which may contribute to medial femoral cortex overload due to their design and frequent varus positioning [6]. However, there is a considerable paucity of data regarding the incidence of CF in curved short-stem implants.

Compared with cemented stems, one contributing factor for CFs in cementless designs is the increased force required during implantation to achieve the necessary press-fit for initial stability [27]. Appropriate sizing of cementless, metaphyseal-anchored stems significantly reduces the incidence of PPFFs by preventing caudal stem migration with consecutive excessive stress on the medial cortex. Undersized stems are associated with increased micromotion and a “blasting effect” from stem migration, both of which heighten the risk of CF formation [16,28]. Further, undersized stems may contribute to both early stem subsidence, leading to medial cortical overload and an increased risk of CF, as well as late stem migration [28].

Interestingly, while CFs are more commonly associated with anatomical stems, classic Vancouver type B fractures occur more frequently in patients with straight-stem designs [11]. Despite the general assumption that CFs predominantly occur intraoperatively or in the early postoperative period, Cottino et al. demonstrated a significant correlation between femoral stem geometry (straight vs. anatomical) and the occurrence of CFs up to 12 years postoperatively [11]. In their analysis of 3248 patients (3593 implants), CFs accounted for 27% of fractures in patients with straight stems and 47% in those with anatomical stems. The authors suggest that the increased fracture rate in anatomical stems may be attributable to the fixation pattern and the mismatch between the metaphyseal and diaphyseal regions. The latter might favor the occurrence of a clamshell pattern, and the partially late onset is due to polyethylene wear and bone loss in the anchoring metaphyseal region. Notably, no stable CFs were identified in this cohort, reinforcing the notion that CFs represent unstable lesions [11].

## 5. Therapeutic Strategies for Clamshell Fractures

To determine the optimal treatment strategy, it is essential to differentiate between the rarely observed simple avulsion fracture of the lesser trochanter (type A_LT_) and a Vancouver type B fracture pattern. While avulsion fractures of the lesser trochanter are typically managed conservatively, Vancouver type B fractures require a careful assessment of femoral stem stability, as the distinction between a stable and unstable stem is critical for appropriate treatment planning and outcome. Recent studies have established that CFs can be classified as a new subtype of Vancouver B2 fractures [9,12]. In contrast, Capello et al. postulated that CF may occur under both circumstances, i.e., with well-fixed and loose implants [12]. Therefore, not all CFs necessitate surgical intervention. The authors suggested that a subset of CFs with a stable osseointegrated implant may be managed non-operatively [12,29]. However, according to the authors’ opinion, considering the above-mentioned pathomechanism of this injury pattern, all CFs should be defined as PPFFs with a potentially loose stem until proven otherwise, especially in the case of metaphyseal-anchored implants. Determining femoral stem stability solely based on conventional radiographs is often challenging and may lead to misinterpretation. Radiographic indicators of stem instability include widening of the calcar region and subsidence of the femoral stem. As such, preoperative computed tomography scanning represents a valuable diagnostic tool and is highly recommended to assess the bone–stem interface as well as the precise location and extent of the fracture [30]. Nevertheless, several studies have reported that up to 20% of femoral stems initially assessed as stable according to preoperative imaging were found to be loose during intraoperative evaluation [31,32].

In 2011, van Houwelingen and Duncan proposed that the treatment strategy for CF should be based on both the timing of the fracture and the size of the medial cortical fragment [9]. According to their evaluation, CF occurs as a result of occult intraoperative fracture as well as within the first 6 weeks after implantation due to stem subsidence with medial cortical overload. The therapeutic approach varies depending on whether CFs occur intraoperatively or during the early postoperative period. In cases of non-occult intraoperative CFs, extraction of the femoral stem, followed by cerclage cable fixation and subsequent reinsertion of the stem, has been suggested as an appropriate therapeutic approach [9,33]. However, if the medial fragment is large and stem stability is compromised, distal fixation of a new stem beyond the fracture site is required [9,26]. In the vast majority of postoperative CFs, stem instability is present, and subsequent revision surgery is necessary. Several operative treatment options are available, including open reduction and internal fixation (ORIF) with cerclage wiring or cerclage banding, isolated stem revision without ORIF, or a combined approach involving both ORIF and stem revision to address stem stability and anatomical fracture reduction. Both the VCS and the UCS consider three critical factors (fracture location, implant stability, and bone stock quality) for therapeutic decision making. Taken together, CFs with a loose stem should be managed following the treatment principles for conventional Vancouver B2 fractures [15,17]. In addition, further extramedullary fixation techniques, such as plate osteosynthesis or the use of structural allografts, can be helpful to enhance mechanical stability and support fracture healing [34].

With respect to stem revision, diaphyseal-anchored stems should be preferred over metaphyseal-anchored stems, because there is an increased risk of mechanical instability in the presence of medial cortical defects and concerns regarding the extent of the medial wall fragment. From a biomechanical perspective, Kastner et al. concluded that in intraoperative CFs using diaphyseal anchored stems, axial stability is maintained if the medial wall defect is not greater than 40% of the stem anchorage. According to the results of their cadaver study, these injuries can be managed with distal cerclage wiring alone [26]. In our opinion, additional medial wall fragment fixation (e.g., via cerclage osteosynthesis) should be considered due to the potential positive impact on functional outcome [7,35]. Individualized treatment planning remains essential to optimize functional outcomes while preserving implant stability [36,37].

Selecting the most appropriate treatment strategy for PPFFs, i.e., whether to perform an isolated open reduction and internal fixation, stem revision alone, or a combination of both, remains a challenging decision. A recent meta-analysis comparing various treatment modalities for Vancouver B2 fractures demonstrated that internal fixation without stem revision is associated with poor clinical outcomes and a significantly higher reoperation rate [38]. These findings align with the recommendations by Huang et al., who advocated for the use of a longer, distally fixed stem in combination with ORIF to ensure optimal fracture stabilization and implant longevity in cases of CF with compromised stem stability [15].

## 6. Limitations

Nevertheless, the current review has some limitations, particularly due to the limited available data on CFs as an acknowledged injury pattern. Additionally, therapeutic outcomes in relation to different treatment approaches remain unclear and should further be investigated. Lastly, our manuscript is prone to all the inherent weaknesses of a narrative review.

## 7. Conclusions

For many years, clamshell fractures were underestimated and not adequately addressed in conventional classification systems for PPFFs. However, with their recent inclusion in the UCS, awareness and understanding of clamshell fractures have improved, leading to better recognition, more accurate diagnosis, and the development of evolving therapeutic strategies. Distinguishing CFs from a simple bony avulsion of the lesser trochanter is crucial, given the differing therapeutic guidelines for each condition. Clamshell fractures should initially be treated as proximal PPFFs associated with an unstable stem, unless proven otherwise. We concur with the prevailing view that CFs typically originate as occult intraoperative injuries that become displaced during the early postoperative period under physiological loading, or as a consequence of primary stem malalignment, leading to caudal migration and metaphyseal cortical failure. Therapeutic options for managing CFs primarily depend on the design and stability of the femoral stem. They may include open reduction and internal fixation, stem revision, or a combination of both. Clamshell fractures with a loose stem should be treated according to the established principles for conventional Vancouver B2 fractures. In many cases, the selected approach is also influenced by the surgeon’s expertise and individual preferences in fracture management.

Ultimately, further studies are necessary to evaluate the clinical outcomes of CFs based on various therapeutic approaches.

The take home messages are as follows:

(1) According to the increasing number of total hip replacements, PPFFs are gaining growing attention among orthopedic surgeons.
(2) Clamshell fractures may occur intra- and postoperatively and are subject to specific patient and implant related risk factors.
(3) Therapeutic options for managing CFs primarily depend on the design and stability of the femoral stem. They may include open reduction and internal fixation, stem revision, or a combination of both.
(4) Clamshell fractures with a loose stem should be treated according to the established principles for conventional Vancouver B2 fractures.
(5) Future research should focus on distinct stem designs and the respective evaluation of associated fracture patterns.

## Abbreviations

CF	Clamshell fracture
ORIF	Open reduction and internal fixation
PPFF	Periprosthetic femoral fracture
THA	Total hip arthroplasty
UCS	Unified Classification System for periprosthetic femoral fractures
VCS	Vancouver classification system
